# A cell-based, quantitative and isoform-specific assay for exchange proteins directly activated by cAMP

**DOI:** 10.1038/s41598-017-06432-4

**Published:** 2017-07-24

**Authors:** Yingmin Zhu, Fang Mei, Pei Luo, Xiaodong Cheng

**Affiliations:** Department of Integrative Biology and Pharmacology, Texas Therapeutics Institute, University of Health Science Center, Houston, Texas USA

## Abstract

Extensive functional studies of the exchange protein directly activated by cAMP (EPAC) family of signaling molecules have demonstrated that EPAC proteins play a fundamental role in several physiological and pathophysiological responses, therefore are attractive drug targets. In this report, the development of a cell-based, medium to high throughput screening assay that is capable of monitoring EPAC-mediated activation of cellular Rap1 in an isoform-specific manner is described. This assay adapts a conventional ELISA format with immobilized RalGDS-RBD as a bait to selectively capture GTP-bound active Rap1. As a result, it fills an urgent need for a cell-based EPAC assay that can be conveniently performed using microtiter plates for the discovery and/or validation of isoform-specific EPAC agonists and antagonists.

## Introduction

Exchange proteins directly activated by cAMP (EAPC1 and EPAC2) mediate the intracellular functions of cAMP by acting as guanine nucleotide exchange factors for the Ras-like small GTPases, Rap1 and Rap2^1, 2^. Extensive studies have demonstrated that EPAC proteins are important signaling molecules involved in modulating a myriad of physiological functions^3–5^, ranging from energy homeostasis^6–8^ and insulin secretion^9, 10^ to learning and memory^11, 12^. In addition, EPAC proteins have also been shown to act as major stress-response mediators and play key roles in the development of human diseases, such as cancer^13–16^, chronic pain^17–19^, cardiovascular diseases^20, 21^ and infection^22, 23^. Therefore, developing small molecule EPAC-specific modulators has evolved into an active area of research within the field for the last few years^24–26^. Several recent studies have reported the effective development of EPAC-specific antagonists using high throughput screening (HTS) biochemical assays^27–29^. However, a robust cell-based assay equipped to measure the activity of EPAC proteins in a medium to high throughput setup is lacking. In this study, the design and execution of an isoform specific cell-based assay capable of measuring cellular activity of EPAC proteins in a microplate format are described.

## Results

### Assay design

**Figure 1:**
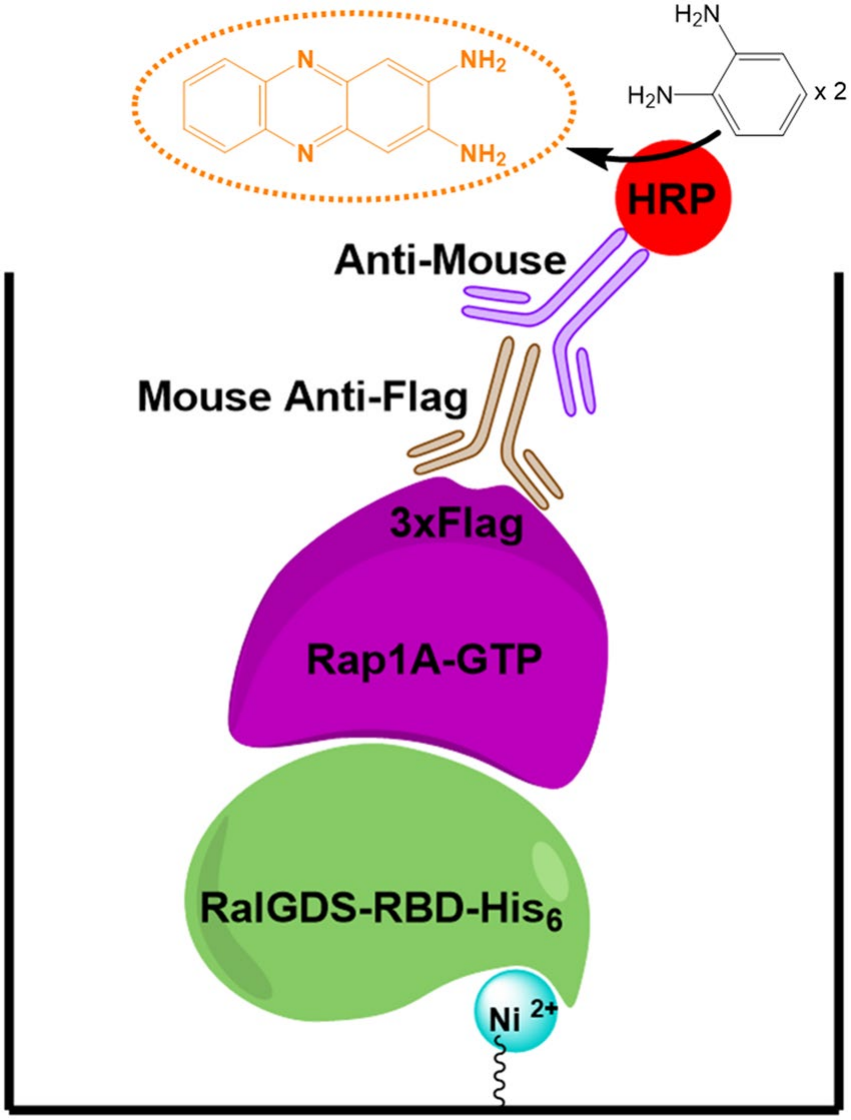
Schematic description of an enzyme-linked immunosorbent assay for Rap1-GTP. Cellular Flag-tagged Rap1-GTP proteins are captured by nickel-immobilized RalGDS-RBD in a well of a microtiter plate and detected by a horseradish peroxidase (HRP)-linked immunosorbent assay using anti-Flag antibodies.

To develop a cell-based, isoform-specific EPAC activation assay, HEK293 cell lines stably expressing Flag-tagged Rap1A and full-length EPAC1 or EPAC2 are proposed. HEK293 cells express minimal endogenous levels of EPAC1 and EPAC2 and have been used as host cells for evaluating the cellular activities of ectopically expressed EPAC proteins in an isoform-specific manner^28^. Activation of EPAC1 or EPAC2 in these cell lines by cAMP elevating agents leads to the accumulation of Flag-Rap1-GTP, which can be captured by RalGDS-RBD immobilized in a nickel-coated 96-well microplate. The levels of active Flag-Rap1-GTP protein can be quantitatively monitored using a specific anti-FLAG antibody and a HRP conjugated secondary antibody in a manner similar to that of a conventional ELISA assay (Fig. 1).

### Assay optimization

**Figure 2:**
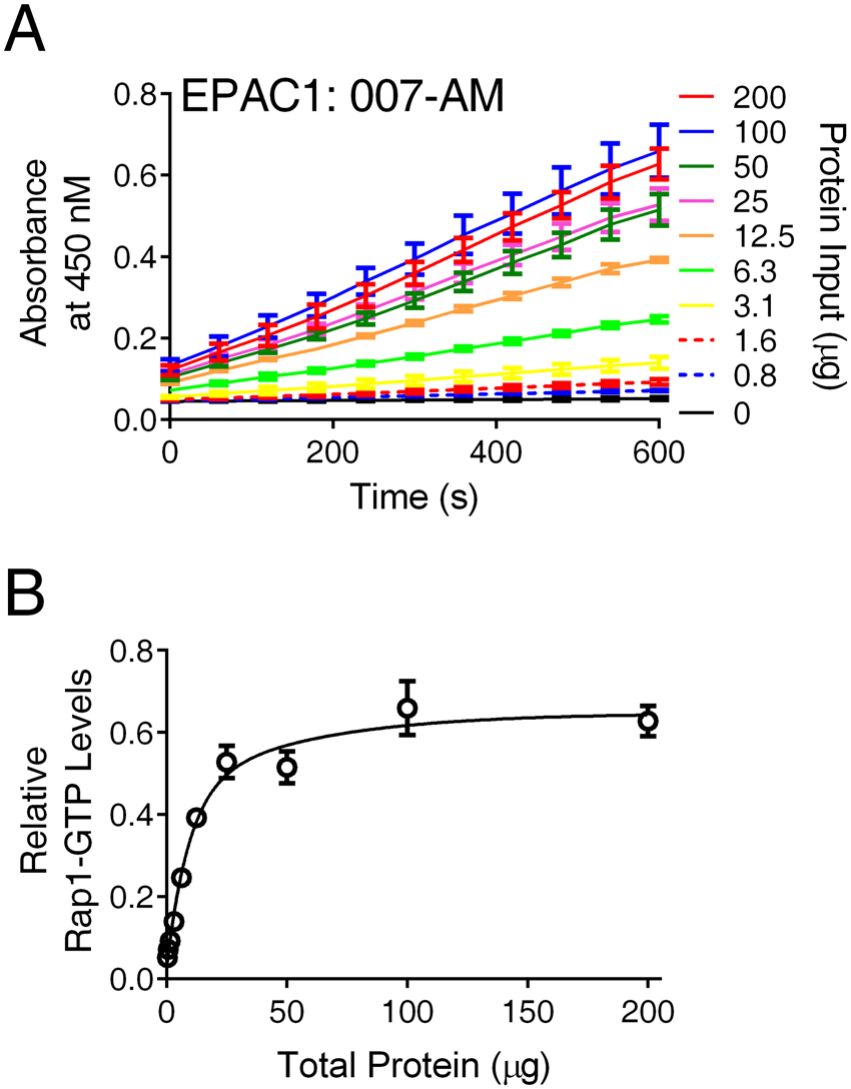
Relative signals of EPAC1-mediated Rap1 cellular activation as function of amount of lysate proteins. (**A**) Time course of EPAC1-mediated Rap1 activation in response to 1 µM of 007-AM measured using various amount lysate protein inputs (0–200 µg). (**B**) Relative measured Rap1-GTP levels as a function of added lysate proteins. Data are presented as Means ± SD, n = 2.

Ectopic expression of  EPAC and Rap1 proteins could lead to elevated basal Rap-GTP levels, which would decrease signal-to-noise ratio and dynamic range of our proposed assay. To optimize the assay, individual stable clones of HEK293/hEPAC1/Flag-Rap1 or HEK293/mEPAC2/ Flag-Rap1 were first established and tested for their basal Rap1-GTP activates. Overall, HEK293/mEPAC2/ Flag-Rap1 cells had higher basal Rap1-GTP activates than their hEPAC1 counterparts, and the basal activities varied within a range of 3-fold among different stable clones. Stable clones with lowest basal activity for HEK293/hEPAC1/Flag-Rap1 or HEK293/mEPAC2/ Flag-Rap1 were selected, expanded and used for subsequent studies. After minimizing the basal Rap1-GTP activates, the levels of total lysate proteins that were optimal to be used for the assay were further determined by testing the signal response as a function of total lysate proteins applied in the assay. As shown in Fig. 2, above background signal could be detected with as little as 0.8 µg total proteins from HEK293/hEPAC1/Flag-Rap1 cell lysate stimulated by1 µM of 8-pCPT-2-O-Me-cAMP-AM (007-AM), a membrane permeable EPAC-specific agonist^30^. The signal readouts were roughly a linear function of the total protein applied up to 25 µg. Therefore, 20 µg total lysate proteins were used for subsequent assay development and optimization.

### Dose-dependent activation of EPAC1 and EPAC2

**Figure 3:**
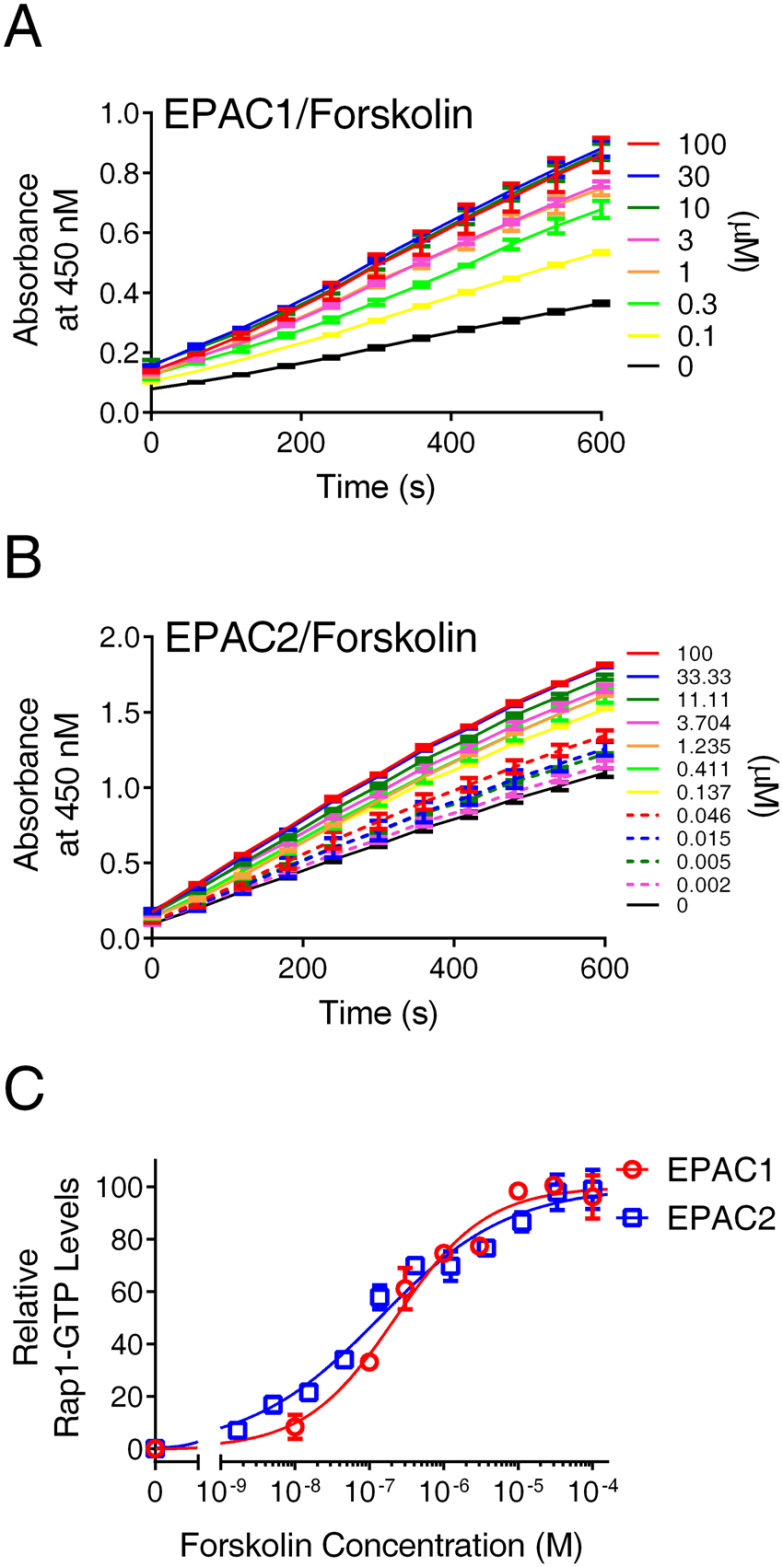
Dose-dependent cellular activation of EPAC1 and EPAC2 by forskolin. (**A**) Time course of EPAC1-mediated Rap1 activation in response to forskolin stimulation. (**B**) Time course of EPAC2-mediated Rap1 activation in response to forskolin stimulation. (**C**) Relative Rap1-GTP levels as a function of forskolin concentration in HEK293 cells overexpressing EPAC1 or EPAC2, respectively. Data are presented as Means ± SD, n = 2.

Forskolin is a labdane diterpene natural product, commonly used a biochemical tool to stimulate intracellular levels of cAMP^31^. Treatment of HEK293/hEPAC1/Flag-Rap1 or HEK293/mEPAC2/ Flag-Rap1 cells with forskolin led to a dose-dependent increase in Rap1-GTP levels in both cell lines (Fig. 3A,B). Forskolin activated HEK293/hEPAC1/Flag-Rap1 or HEK293/mEPAC2/ Flag-Rap1 cells with similar potencies with apparent half maximal activation constants (AC_50_) of 226 ± 57 and 137 ± 36 nM, respectively (Fig. 3C). Using a conventional affinity pull-down assay^32^, a similar dose-dependent activation of Rap1 by forskolin in HEK293/hEPAC1/Flag-Rap1 cells was observed (Supplemental Fig. 1). Since forskolin-mediated increase in intracellular cAMP activates both EPAC and PKA, to ensure that the signals observed in our assay were not in part due to the activation of PKA, control experiments in the presence of a PKA-specific inhibitor, H89, were performed. As shown in Supplemental Fig. 2, the effects of 10 µM H89 on both basal and forskolin-induced Rap1 activities were minimal, suggesting the readouts observed in HEK293/hEPAC1/Flag-Rap1 or HEK293/mEPAC2/ Flag-Rap1 cells in response to forskolin activation were mediated by EPAC1 or EPAC2, respectively.

**Figure 4:**
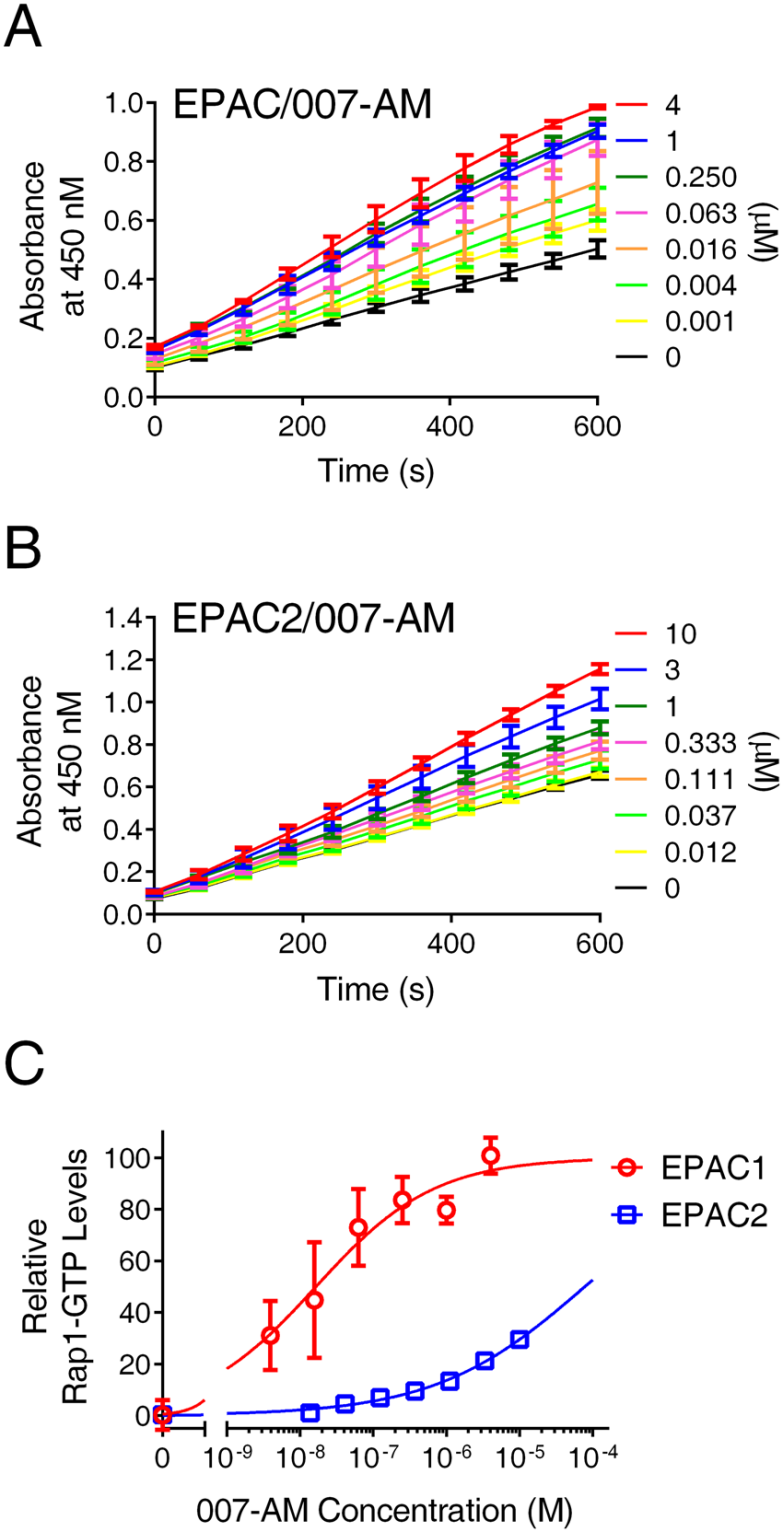
Dose-dependent cellular activation of EPAC1 and EPAC2 by 007-AM. (**A**) Time course of EPAC1-mediated Rap1 activation in response to 007-AM stimulation. (**B**) Time course of EPAC2-mediated Rap1 activation in response to 007-AM stimulation. (**C**) Relative Rap1-GTP levels as a function of 007-AM concentration in HEK293 cells overexpressing EPAC1 or EPAC2, respectively. Data are presented as Means ± SD, n = 3.

To further assess the EPAC-mediated cellular activation of Rap1 in our assay system, dose-dependent experiments using 007-AM were performed. As shown in Fig. 4, 007-AM dose-dependently activated EAPC1 in HEK293/hEPAC1/Flag-Rap1 cells with an apparent AC_50_ value of 16.5 ± 3.1 nM. On the other hand, 007-AM did not activate EPAC2 as robust as EPAC1, the does-dependent activation did not reach plateau even with 10 µM 007-AM in HEK293/mEPAC2/ Flag-Rap1 cells. These observation is consistent with the fact that 007 is a poor activator of EPAC2^26^.

### Pharmacological Inhibition of cellular EPAC1 and EPAC2

**Figure 5:**
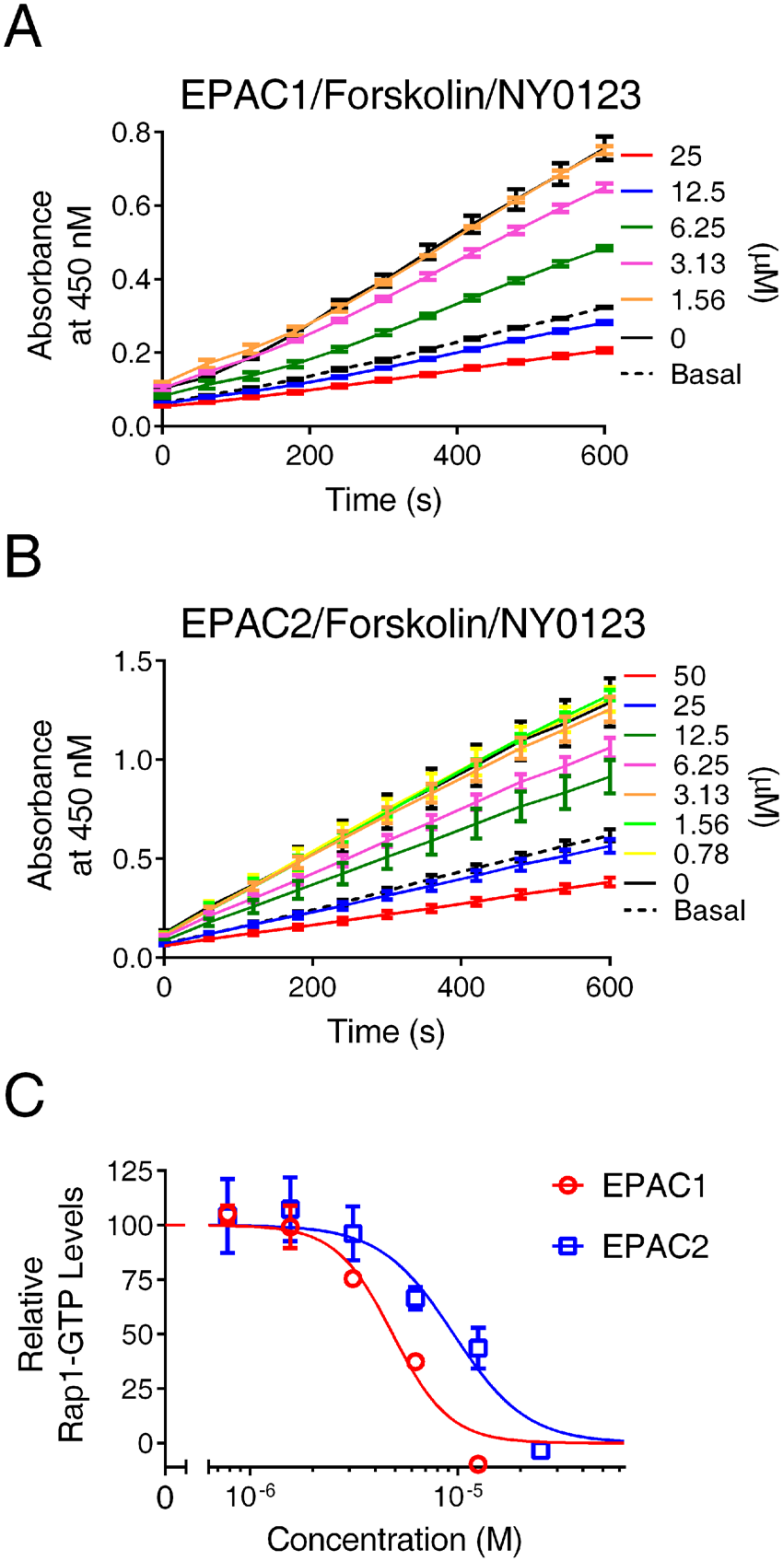
Dose-dependent inhibition of forskolin-induced cellular EPAC1 and EPAC2 activation by NY-0123. (**A**) Time course of EPAC1-mediated Rap1 activation induced by 1 µM Forskolin in the presence of various concentrations of NY-0123. (**B**) Time course of EPAC2-mediated Rap1 activation induced by 1 µM Forskolin in the presence of various concentrations of NY-0123. (**C**) Relative Rap1-GTP levels induced by 1 µM forskolin as a function of NY-0123 concentration in HEK293 cells overexpressing EPAC1or EPAC2, respectively. Data are presented as Means ± SD, n = 3.

To further validate our cell-based EPAC activation assay, the effect of pharmacological inhibition on cellular EPAC activation were examined with NY-0123, an EPAC-specific antagonist that is based on our lead compound ESI-09 and known to inhibit both the EPAC1 and EPAC2 activities^33^. As shown in Fig. 5, pretreatment of HEK293/hEPAC1/Flag-Rap1 or HEK293/mEPAC2/ Flag-Rap1 cells with NY-0123 dose-dependently inhibited cellular EPAC1 and EPAC2 activities stimulated by 1 µM Forskolin with apparent IC_50_ values of 4.9 ± 0.6 and 9.6 ± 1.6 µM, respectively. The testing compound concentrations were kept below 25 µM as it is known that the aqueous solubility of this class of EPAC inhibitors is low and the compound may form aggregates at higher concentrations^34^. In fact, a decreased recovery of total lysate proteins was observed when cells were treated with higher concentrations of NY-0123.

**Figure 6:**
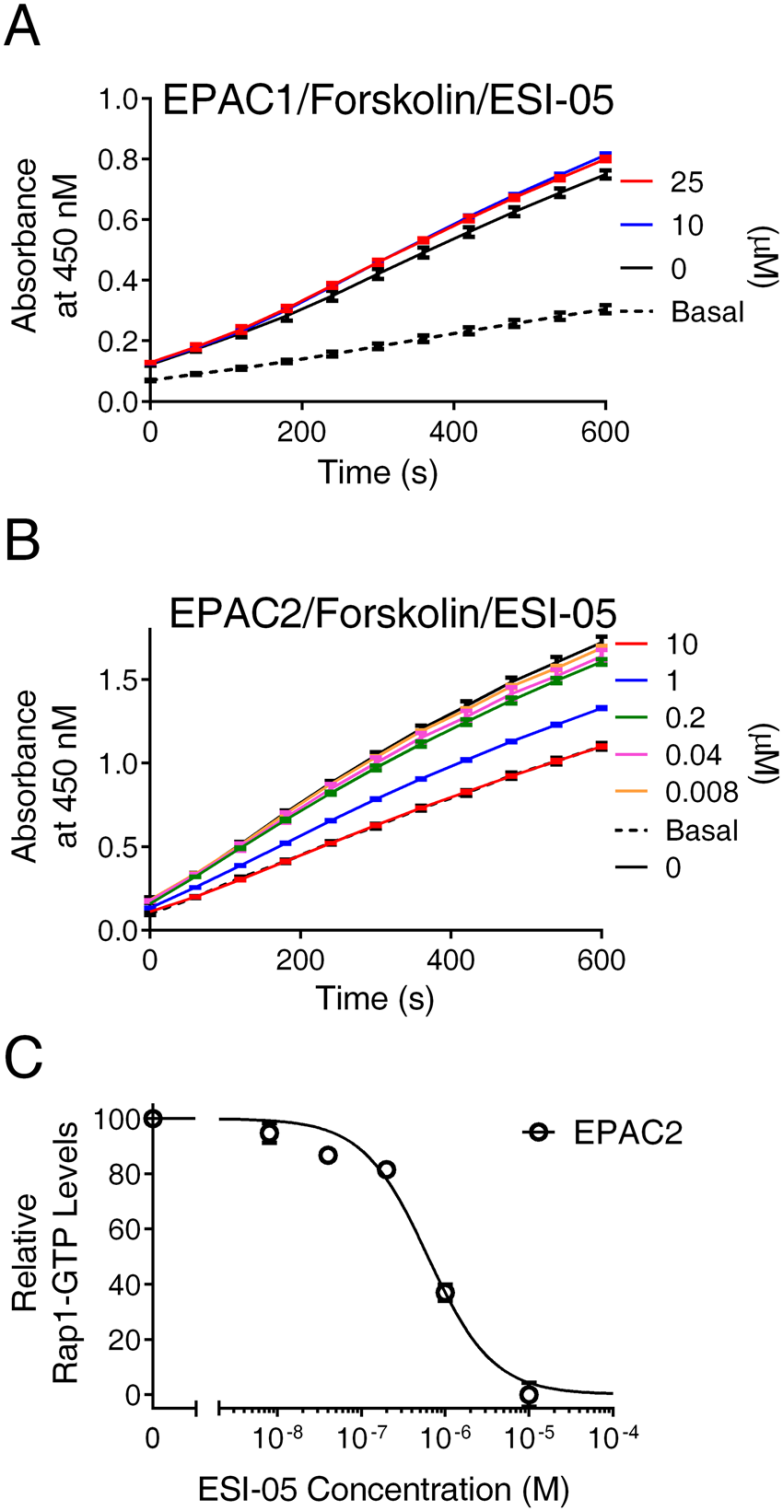
Dose-dependent inhibition of forskolin-induced cellular EPAC1 and EPAC2 activation by ESI-05. (**A**) Time course of EPAC1-mediated Rap1 activation induced by 1 µM forskolin in the presence of various concentrations of ESI-05. (**B**) Time course of EPAC2-mediated Rap1 activation induced by 1 µM forskolin in the presence of various concentrations of ESI-05. (**C**) Relative Rap1-GTP levels induced by 1 µM forskolin as a function of ESI-05 concentration in HEK293 cells overexpressing EPAC2. Data are presented as Means ± SD, n = 2.

To ascertain the isoform-specific capability of our cell-based EPAC activity assay, the effect of an EPAC2-specific inhibitor, ESI-05, on forskolin-mediated cellular EPAC1 and EPAC2 activation was evaluated. ESI-05 selectively inhibited EPAC2, but not EPAC1, with sub-micromolar potency in the presence of 20 µM cAMP^28, 35^. While pretreatment of HEK293/mEPAC2/ Flag-Rap1 cells with ESI-05 dose-dependently inhibited cellular EPAC2 activities stimulated by 1 µM forskolin (Fig. 6A) with an apparent IC_50_ value of 621 ± 154 nM (Fig. 6C), ESI-05 had no detectable activity in inhibiting cellular EPAC1 activation stimulated by 1 µM Forskolin (Fig. 6B). These data not only further confirm that ESI-05 truly is selective for EPAC2 but also validate that our assay indeed allows isoform-specific measurement of cellular EPAC1 and EPAC2 activities.

**Figure 7:**
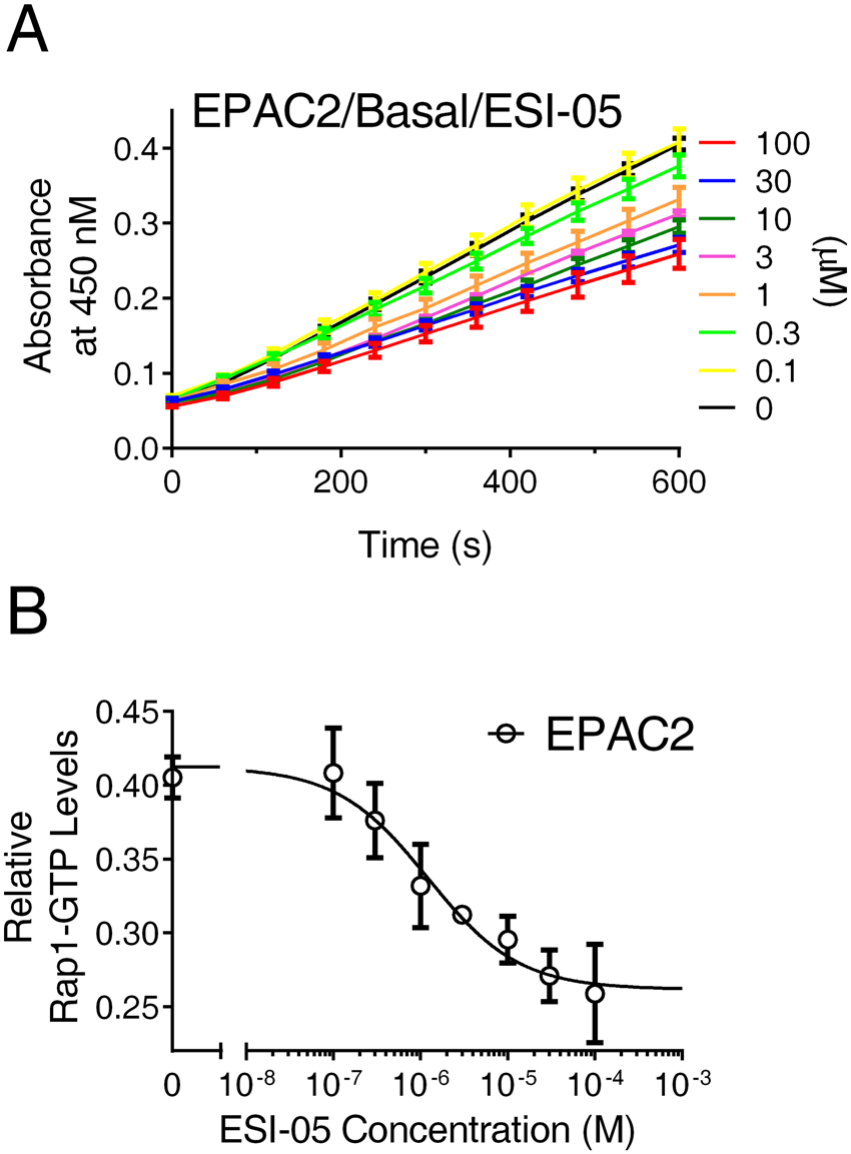
Dose-dependent inhibition of basal EPAC2 activation by ESI-05. (**A**) Time course of EPAC2-mediated basal Rap1 activation in the presence of various concentrations of ESI-05. (**B**) Relative basal Rap1-GTP levels as a function of ESI-05 concentration in HEK293 cells overexpressing EPAC2. Data are presented as Means ± SD, n = 3.

During the course of the studies, it was observed that ESI-05, at high concentrations, is capable of suppressing cellular EPAC activity to a level significantly below the basal activity, suggesting that ESI-05 may act as an apparent inverse agonist for EPAC2. To test this hypothesis, a new series of experiments monitoring the basal activity of HEK293/mEPAC2/Flag-Rap1 cells as a function of ESI-05 were performed. As shown in Fig. 7, ESI-05 does-dependent inhibited basal EPAC2 activity with an IC_50_ value of 1.2 ± 0.4 µM.

## Discussion

Extensive researches have demonstrated that the EPAC proteins play key roles in various physiological and pathophysiological functions and are attractive drug targets^3, 21, 25^. The development of a cell-based assay for the discovery of EPAC-specific modulators has become an important area of research. Conventionally, EPAC cellular activity is assayed by monitoring relative cellular Rap-GTP levels using affinity pull-down with purified recombinant GST-RalGDS-RBD, which preferentially binds Rap-GTP over Rap-GDP with approximate 4,000-fold selectivity^32^. The levels of Rap-GTP from the affinity pull-down are further evaluated using immunoblotting analysis. This approach is laborious, time-consuming, costly, and involves a multi-step procedure prone to operational errors. Furthermore, Western blot analysis is difficult to quantify and not suitable for medium to high throughput experiments. To overcome these obstacles, a cell-based EPAC assay that enables the detection and quantification of EPAC-mediated cellular Rap1 activation under the microplate format has been designed, developed and optimized. In this assay, the immobilization of the purified His_6_-tagged RalGDS-RBD in the nickel-coated well of a 96-well plate allows convenient capture of active Rap1-GTP from the cell lysate, which can be easily monitored and quantified by enzyme-linked immunosorbent detection using a microplate reader.

With proper optimizations, our assay is capable of quantitatively detecting cellular EPAC activation in response to various stimuli as well as inhibitors in a dose-dependent and isoform-specific manner. This new assay has several clear advantages over other existing cell-based EPAC assays. Compared to the traditional bead-based Rap-GTP pull-down assay, the immobilized RalGDS-RBD bait in microplate enables assay miniaturization and automation. In fact, the assay can be performed in parallel for a large number of samples, each with as little as a few micrograms of total lysates. Moreover, if offers more reproducible and consistent capture of the Rap-GTP protein in the lysate and allows more convenient and reliable signal detection and quantification. By adapting a microplate format, our assay eliminates the need of centrifugation washing steps and immune-blotting based detection. Hence, the entire assay can be performed within hours, instead of days. Another alternative cellular EPAC assay utilizes EPAC-based fluorescence resonance energy transfer (FRET) or bioluminescence resonance energy transfer (BRET) sensors. These genetically engineered sensors can be ectopically expressed in various cells for the detection of generation of intracellular cAMP in live cells in a spatial and temporal manner^36–38^. While cells stably expressing these biosensors can be adapted for microplate format assays, the signal-to-noise and dynamic range of these biosensors are normally poor and not adequate for HTS. Importantly, positive FRET or BRET signals driven by conformational changes of EPAC proteins do not always correlate with EPAC activation or inhibition, and conversely allosteric modulators of EPAC may not necessarily induce conformational changes that affect FRET/BRET-based readout. These drawbacks limit the general applicability of using EPAC-based biosensors for functional cell-based EPAC assays.

During the development of the assay, it became apparent that ectopic expression of EPAC and Rap1 raised the basal level of active Rap1, which decreased signal-to-noise ratio and dynamic range of the assay. In particular, HEK293/mEPAC2/Flag-Rap1 cells had higher baselines of active Rap1-GTP as compared those of HEK293/hEPAC/Flag-Rap1 cells. This is most likely due to the fact that EPAC2 protein has a more than three-fold higher affinity for cAMP than EPAC1 protein. Through careful selection of cell clones with low levels EPAC and Rap1 expression, suitable signal-to-noise ratio and dynamic range could be achieved. Ultimately, the use of an inducible expression system or genomic knock-in may provide a better solution of solving the aforementioned high basal activity problem. Another alternative is to use the endogenously expressed Rap1 protein as the readout, however, the absence of highly specific and sensitive Rap1 antibodies prevents its execution as currently available anti-Rap1 antibodies lack appropriate specificity and sensitivity for monitoring endogenous Rap1 activation using the microplate format.

While the assay described in this study is specifically designed for Rap regulators EPAC1 and EPAC2, Rap small GTPases have multiple regulators, including C3G, PDZ-GEF1 and 2, CalDAG-GEF1, Dock4 and RapGAPs^39^. In theory, depending upon their cellular expression, these Rap modulators may interfere with the assay. However, considering that the design of the assay involves ectopic expression of EPAC proteins and their selective activation using cAMP stimulating agents, the possibility of interference by these non-EPAC Rap modulators should be minimal and negligible.

In summary, a sensitive cell-based EPAC activity assay that can be monitored using a microplate reader has been established. This assay can be performed to screen and/or validate EPAC isoform-specific agonists or antagonists in medium to high throughput formats, therefore, represents a useful tool for the discovery EPAC-based pharmacological probes.

## Material and Method

### Reagents

Tween-20, IGEPAL CA-630 (molecular biology grade), o-phenylenediamine dihydrochloride (OPD) tablets (#P3804), MEM medium (#M4655) and anti-FLAG M2 monoclonal antibody (#F1804) were purchased from Sigma. Protein inhibitor cocktail tablets, EDTA free (#88666), Nickel Coated Plates, Clear, 8-Well Strip (#15142) were obtained from Thermo Scientific. DC protein assay kit (#5000112) and Goat Anti-Mouse IgG HRP-Conjugated (#170–6516) were from Bio-Rad. Thrombin (#27084601), Glutathione Sepharose 4B resin (#17075605) and HiLoad Superdex 200 16/600 column (#28989335) were the products from GE Healthcare. All other chemicals were reagent grades.

### Recombinant Protein expression and purification

N-terminal GST-tagged and C-terminal His_6_-tagged wild-type rat Rap binding domain (RBD, aa 726–823) of Ral GDS protein (GST-RalGDS-RBD-His_6_) and its inactive triple-alanine mutant (R784A, K796A and K805A)^30^ were constructed in the pGEX-2T vector. GST-RalGDS-RBD-His_6_ proteins were expressed in *E.coli* BL21 (DE3) and isolated using the Glutathione Sepharose 4B resin with a standard GST-fusion protein purification protocol in a buffer containing 20 mM Tris-HCl, 150 mM NaCl, pH 7.4. Thrombin-cleaved RalGDS-RBD-His_6_ proteins were further purified using a HiLoad Superdex 200 16/600 size exclusion column, which was connected to a Bio-Rad NGC chromatography system.

### Cell lines

HEK293 (ATCC) cells were grown in MEM medium supplemented with 10% heat-inactivated FBS (Invitrogen), at 37 °C, 5% CO_2_. HEK293 stable cell lines expressing Flag-tagged human Rap1A (Flag-hRap1A) alone and co-expressing human EPAC1 (hEPAC1) and Flag-hRap1A or mouse EPAC2 (mEPAC2) and Flag-hRap1A were established by sequential transfections using Lipofectamine 2000 reagent (Invitrogen) with mammalian expression vectors, first with pIRES2-EGFP containing Flag-hRap1A and followed by pIRES‐hyg2 containing hEPAC1 or mEPAC2. Individual stable cell clones were selected and maintained in MEM containing 500 µg/ml G418 for pIRES2-EGFP and 100 µg/ml hygromycin B for pIRES‐hyg2, respectively.

### Cellular Rap1 assay

HEK293 cells co-expressing hEPAC1/Flag-tagged hRap1A or mEPAC2/Flag-tagged hRap1A were seeded in 12-well plates (Corning #3513) at an initial confluency of 400,000 cell/well and incubate at 37 °C, 5% CO_2_, in MEM medium with 10% FBS, for 24 hours. Cells were then rinsed with HBSS and cultured in FBS-free MEM medium for another 16 hours. If necessary cells were first pretreated with various concentration of antagonists or vehicle at 37 °C for 30 min. Otherwise, the cells were stimulated with various concentrations of agonists or vehicle controls at 37 °C for 10 min, then immediately rinsed with ice-cold PBS and lysed in 500 µl ice-cold lysis buffer containing 20 mM Tris-HCl, pH 7.4, 150 mM NaCl, 10 mM MgCl_2_, 1% IGEPAL CA-630 and proteinase inhibitors cocktail. Cell extracts were clarified by centrifugation at 18,000 RCF for 20 min and their protein concentrations were determined by DC protein assay kit. 100 µl of properly diluted cell lysates containing 0 to 50 µg total proteins were added to nickel coated 96-well plate strips that were pre-coated with 200 µg of RalGDS-RBD-His_6_ proteins and incubated at 4 °C for 2 hours with gentle rocking. After washing with 100 µl ice-cold ELISA buffer (20 mM Tris-HCl, pH 7.4, 150 mM NaCl, 10 mM MgCl_2_ and 0.1% Tween-20) for 3 times, 100 µl mouse anti-Flag antibody (1:5000 in ELISA buffer) was added into each well and incubated at 4 °C for 1 hour with gentle shaking. Following three washes with 100 µl ice-cold ELISA buffer, 100 µl anti-mouse HRP-conjugated antibodies (1:3000 in ELISA buffer) were added into each well. After incubation at room temperature for 1 hour with gentle shaking, the wells were washed three times with 100 µl ELISA buffer. 200 µl of OPD substrate was added into each well and absorbance signal at 450 nm was monitored using an M2 microplate reader (Molecular Devices) for 30 min with 1-min intervals. 10-min data points were used for quantification.

### Data availability statement

All data generated or analyzed during this study are included in this published article and its Supplementary Information file.

## Electronic supplementary material

Supplementary Figures
