# Supplementary material for: A cell-based, quantitative and isoform-specific assay for exchange proteins directly activated by cAMP
Source: Sci Rep. 2017 Jul 24;7:6200. doi: 10.1038/s41598-017-06432-4 (PMC5524698; doi:10.1038/s41598-017-06432-4)
Supplement: Supplementary file 1 — Supplementary Figures [file 41598_2017_6432_MOESM1_ESM.doc]

**A cell-based, quantitative and isoform-specific assay for exchange proteins directly activated by cAMP**

Yingmin Zhu, Fang Mei, Pei Luo and Xiaodong Cheng*

Department of Integrative Biology and Pharmacology, Texas Therapeutics Institute, University of Health Science Center, Houston, Texas, USA

*To whom correspondence should be addressed:

Xiaodong Cheng, PhD.

Department of Integrative Biology and Pharmacology, The University of Health Science Center, 6431 Fannin Street, Houston, Texas 77030-1051

Tel.: (713)500-7487

Fax: (713)500-7456

Email: [xiaodong.cheng@uth.tmc.edu](mailto:xiaodong.cheng@uth.tmc.edu).

**
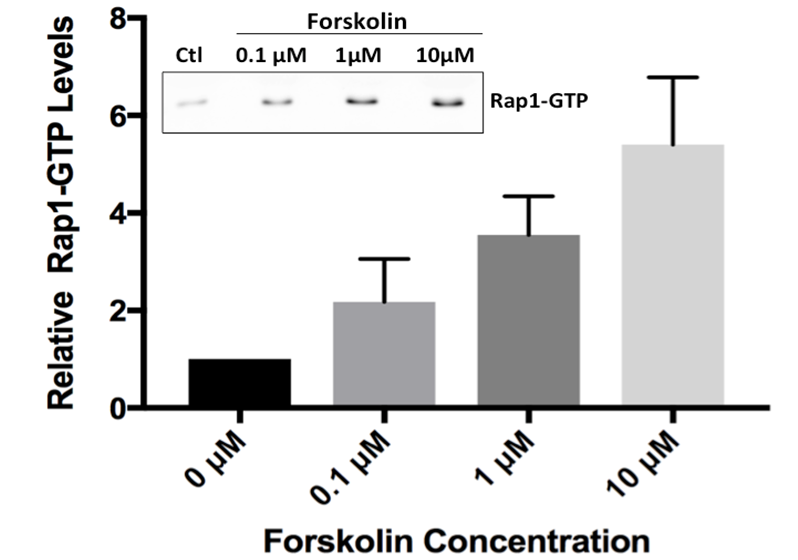
**

**Figure S1. Dose-dependent cellular activation of EPAC1 by forskolin.** Relative Rap1-GTP levels, as monitored by affinity pull-down assay using GST-RalGDS-RBD, in HEK293 cells overexpressing EPAC1 stimulated by various concentrations of forskolin. Inset: representative immunoblot image of Rap1-GTP levels under basal and forskolin-stimulated conditions.


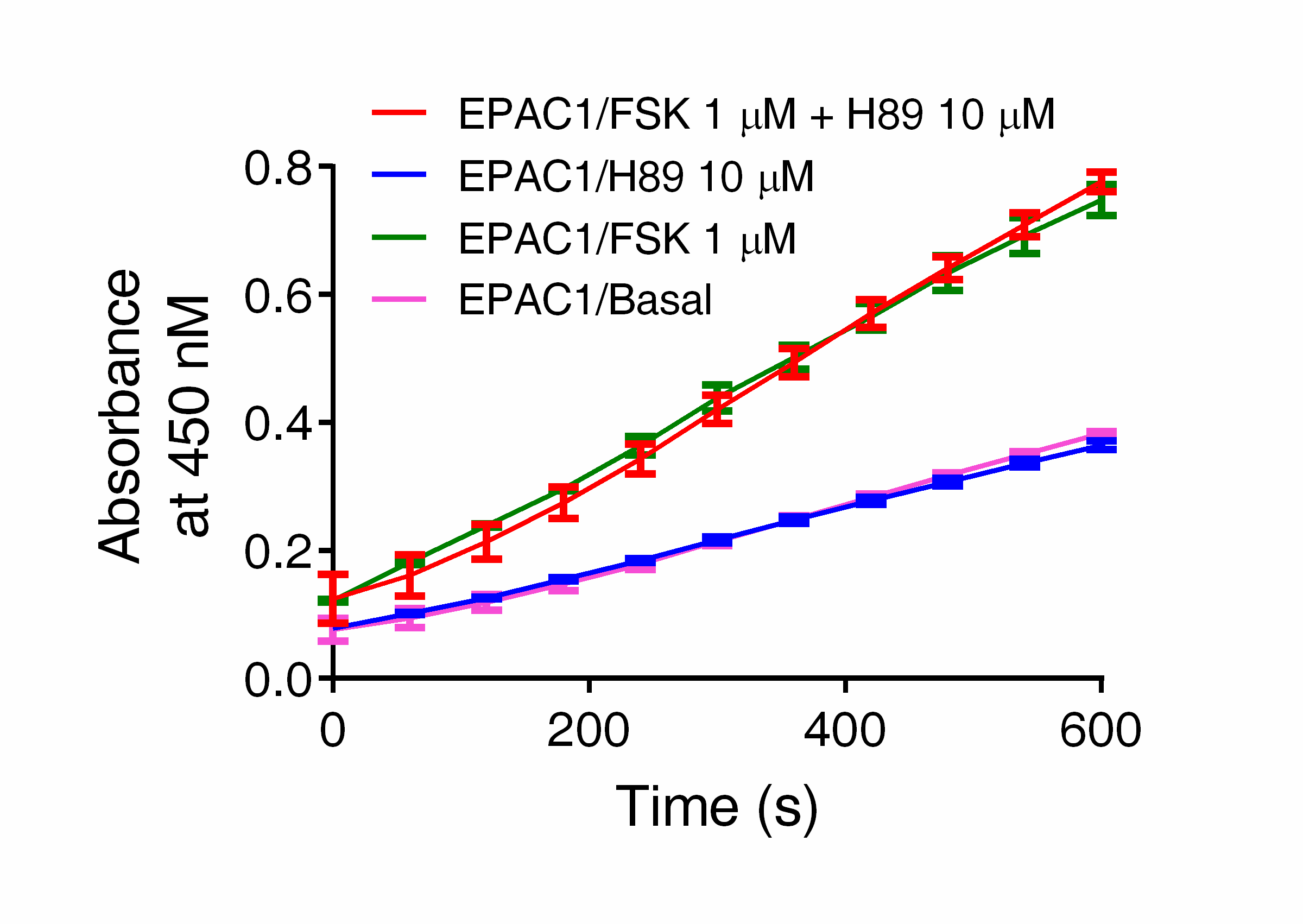
**Figure S2. Effect of H89 on basal and forskolin-stimulated Rap1 activities.** Time course of EPAC1-mediated Rap1 activation under basal or forskolin-stimulated conditions in the presence or absence of H89 in HEK293 cells overexpressing EPAC1.
